# Tumor-selective Blockade of CD47 Signaling with CD47 Antibody for Enhanced Anti-tumor Activity in Malignant Meningioma

**DOI:** 10.2174/1570159X21666230511123157

**Published:** 2023-08-15

**Authors:** Xiaotong Liu, Huarong Zhang, Chaohu Wang, Zhiyong Li, Qianchao Zhu, Yiwen Feng, Jun Fan, Songtao Qi, Zhiyong Wu, Yi Liu

**Affiliations:** 1 The First School of Clinical Medicine, Southern Medical University, Guangzhou, Guangdong, China;; 2 Department of Neurosurgery, Nanfang Hospital, Southern Medical University, Guangzhou, Guangdong, China;; 3 College of Traditional Chinese Medicine, Southern Medical University, Guangzhou, Guangdong, China

**Keywords:** Malignant meningioma, CD47, immune escape, EMT, targeted therapy, macrophages

## Abstract

**Background:**

Patients with WHO grade III meningioma have a poor prognosis with a median survival of less than two years and a high risk of recurrence. However, traditional treatment options have failed to improve prognosis. Therefore, development of novel immunotherapy targets is urgently needed. CD47 acting as a “don't eat me” signal to macrophages can trigger tumor immune escape. However, the role of CD47 in malignant meningioma is not well understood.

**Methods:**

We collected 190 clinical meningioma samples and detected the expression of CD47 and immune infiltration in WHO grade I-III by immunohistochemistry, western blot, qPCR. We also examined the functional effects of anti-CD47 on cell proliferation, migration and invasion, macrophage-mediated phagocytosis and tumorigenicity both *in vitro* and *in vivo*.

**Results:**

We found that the expression of CD47 was increased in malignant meningioma along with a decreased number of T cells and an increase in CD68^+^ macrophages. Blocking CD47 with anti-CD47 antibody (B6H12) suppressed tumor cell growth, motility and promoted macrophage-mediated phagocytosis in IOMM-Lee cells *in vitro*. *In vivo* experiments showed that anti-CD47 antibody (B6H12 or MIAP301) significantly inhibited the tumor growth and this effect was partly blocked by the depletion of macrophages. Finally, p-ERK and EGFR showed higher expression in malignant meningioma with high expression of CD47, which was verified by western blot.

**Conclusion:**

Our results demonstrated that CD47 maybe involved in the meningioma progression and prognosis and offered a novel therapeutic option by targeting CD47 in malignant meningioma.

## 1 INTRODUCTION

Meningioma accounts for up to 30% of all primary intracranial tumors. The prognosis of meningioma is closely related to the pathological classification. They are histologically classified into three types according to the World Health Organization (WHO) classification of tumors [1]. Approximately 3% variants correspond with WHO grades III [2], which presents a worse prognosis with a higher risk of recurrence revealing a 5-year mortality rate of up to 50-80% and shorter median survival times of less than two years.

However, traditional treatments such as surgical resection, chemotherapy have difficulty in treating malignant meningioma effectively [3]. Recently, immune therapies have showed impressive effects in various tumors [4]. Programmed death-ligand 1 (PD-L1), was shown to trigger immune escape in malignant meningioma [5]. Meanwhile, CD47 as an immunoglobulin family member protein overexpressed in various cancers [6]. CD47 acts as a “don’t eat me” signal by binding to signal regulatory protein α (SIRPα) on macrophages and thereby inhibiting phagocytosis of target cells. Previous studies have proved this ability of CD47 [7-9]. Meanwhile, anti-CD47 antibodies have been confirmed as an inhibitor to cancer cell proliferation through the EGFR/PI3K/AKT signaling pathway [10-12]. And it has been proved that CD47 blockade can also enhance tumor cell phagocytosis by tumor-associated macrophages (TAMs) [13]. However, the role of CD47 in tumor immunity has not been studied in malignant meningioma.

In light of these previous findings, we collected 190 meningioma samples to assess the expression of CD47 and the infiltration of immune cells. Moreover, we conducted *in vitro* and *in vivo* experiments to find whether anti-CD47 antibody could suppress the proliferation of malignant meningioma and promote the macrophage-mediated phagocytosis. Our results showed that CD47 was highly expressed in meningioma and was closely related to the prognosis of meningioma, and macrophages were the major types of the immune cells in meningioma compared with other immune cells. Furthermore, *in vitro* experiments indicated that CD47 could enhance the cell growth, migration and invasion of IOMM-Lee cells and CD47 blockade could promote the phagocytosis of malignant meningioma cells by macrophages. Finally, *in vivo* experiments showed that anti-CD47 antibody (B6H12 or MIAP301) significantly inhibited the tumor growth and this effect of inhibition was partly blocked by the depletion of macrophages. The molecular pathway analysis suggested that the expression of p-ERK and EGFR was up-regulated by CD47. In sum, our results indicated that CD47 maybe serve as a potential therapeutic target of meningioma.

## 2 MATERIALS AND METHODS

### 2.1 Patients and Tissue Samples

We collected a total of 190 cases of formalin-fixed and paraffin-embedded (FFPE) meningioma tissues from Department of Neurosurgery, Nanfang Hospital. The cohort included 190 meningioma patients with a median age of 51 years (range, 13-82 years) including 68 male and 122 female patients with 128 WHO grade I, 37 WHO grade II and 25 WHO grade III. They were all cases of meningioma diagnosed by the Department of Pathology of Nanfang Hospital from 2008 to 2019. Fresh meningioma tissue samples for primary cell culture were obtained from surgery and all patients provided written informed consent prior to acquisition of tissue. The above specimens were examined and identified by three board certified surgical pathologists specializing in meningioma pathology, and were reclassified according to the 2007 WHO classification of meningioma. Details of the patient information were presented in Table **1**.

### 2.2 Cell Lines

The human malignant meningioma cell line IOMM-Lee (RRID: CVCL_5779) was cultured in complete medium, specifically Dulbecco's Modified Eagle Medium (DMEM, PAA) with 10% fetal bovine serum (FBS, Invitrogen) and 1% penicillin/streptomycin (Invitrogen) at 37°C with 5% CO_2_. All human cell lines have been authenticated using STR (or SNP) profiling within the last three years has been included. All experiments were performed with mycoplasma-free cells.

### 2.3 Antibodies

PD-L1 (1:400; Cell Signaling Technology; #13684), CD47 (1:200; Sigma; HPA044659), GAPDH (1:2000; Cell Signaling Technology; #5174), CD3 (1:200; Thermo Fisher; 14-0038-80), CD4 (1:150; Thermo Fisher; 14-0041-82), CD8 (1:100; Thermo Fisher; 14-0081-82), SIRPα (1:200; Abcam; ab267409), CD20 (1:100; Abcam; ab78237), FoxP3 (1:300; Abcam; ab20034), Ki-67 (1:200; Abcam; ab16667), EGFR (1:50; Cell Signaling Technology; #2085), p-ERK (1:400; Cell Signaling Technology; #3074), total-ERK (1:500; Cell Signaling Technology; #4695), β-catenin (1:200; Abcam; ab32572), CD68 (1:100; Abcam; ab213363), EMA (1:300; Abcam; ab109185), Anti-CD47 antibody (Clone: MIAP301) (100μg/day *in vivo*; BioXcell), Anti-CD47 antibody (Clone: B6H12) (10μg/mL *in vitro* and 100μg/day *in vivo*; BioXcell), isotype control (mouse IgG1, BioXcell).

### 2.4 Immunohistochemistry

HRP/DAB Detection IHC Kit (Abcam) was used for the following steps according to the manufacturer's protocol. All samples were assessed by three pathologists in ten different high-power fields (HPFs). The staining intensity of CD47 and PD-L1 was scored as 0 (no staining), 1 (weak staining), 2 (intermediate staining), or 3 (dark staining). The percentage of staining cells was scored as 0 (0-5%), 1 (6-25%), 2 (26-50%), 3 (51-75%), or 4 (76-100%). The product of the two scores was considered as the final score. A composite score greater than the median value for all cases was considered ‘high expression’ [14]. The histoscore of the membrane and nuclear staining quantification was assessed according to the formula (3+ per cent cells) × 3+(2+ per cent cells) × 2+(1+ per cent cells) × 1, and the formula total intensity/total cell number was used to assess the histoscore of pixel quantification. In this case, the normalized score is between 0 and 250 [15]. The χ^2^ test was used to analyze the immune checkpoint expression rate in different grades of meningioma.

### 2.5 Mice

A total of 18 nude mice (BALB/c) were provided and raised by the Experimental Animal Center of Nanfang Hospital of Southern Medical University. The mice were 4 weeks old and weighed 20-25g. All animals were maintained on a 12-h light/dark cycle at 23°C with food and water available *ad libitum*. All animal studies were performed according to the National Institutes of Health Guide for the Care and Use of Laboratory Animals and approved by the Experimental Animal Committee of the Nanfang Hospital, Southern Medical University.

### 2.6 Instruments and Reagents

DMEM medium (Gibco, USA), 0.25% trypsin and 0.05% ethylenediamine tetraacetic acid (EDTA) (Gibco, USA), fetal bovine serum (FBS) (Gibco, USA), phosphate buffer solution (PBS) (Gibco, USA), CO_2_ constant temperature incubator (Thermo Fisher company, USA), PMA (1:10000; Sigma), Transwell chamber (Corning, 12 wells).

### 2.7 Isolation, Culture and Purification of Primary Cells

The fresh meningioma tissue specimens obtained from surgery were washed with PBS for three times and were cut into 1 mm^3^ patches by ophthalmic scissors. The tissue blocks were then digested with 0.25% trypsin for 30 minutes and filtered with 70 μm filter. The filtered solution was centrifuged for 3 minutes at 1000 rpm/min to remove the impurities. Next, 3 ml red blood cell lysis buffer was added into the filtered solution for 3 minutes. Finally, the suspension was diluted with 6 ml PBS and centrifuge was used again to remove the supernatant. Cells were planted in 25 cm^2^ culture bottles with DMEM medium containing 10% FBS and 1% penicillin/streptomycin in 37°C, 5% CO_2_ cell incubator under aseptic conditions. Subculturing was performed when the cell density was greater than 90% about every 2 to 3 days.

### 2.8 Western Blot Analysis

The protein was extracted from malignant meningioma cells. The concentration of protein was determined using a BCA Protein Assay Reagent kit (Pierce; Thermo Fisher Scientific, Inc., Waltham, MA, USA). Protein bands were detected by chemiluminescence technology using an ECL advanced western blot analysis detection reagent (EMD Millipore), quantified and normalized to GAPDH using Image J software (version 1.38, National Institutes of Health, Bethesda, MD, USA).

### 2.9 RNA Extraction and Quantitative PCR

Quantitative PCR (qPCR) was performed using the SYBR Green Master Mix (Takara Bio Inc.) in a 20 μl reaction volume, according to the manufacturer's instructions. All reactions were run in triplicate on Step One™ PCR amplifier (Applied Biosystems), and GAPDH gene was used as endogenous control. The primer sequences for CD47 gene were 5′-AGAAGGTGAAACGATCATCGAGC-3′ (sense primer) and 5′-CTCATCCATACCACCGGATCT-3′ (antisense primer). The primer sequences for GAPDH gene were 5′-ACCACAGTCCATGCCATCAC-3′ (sense primer) and 5′-TCCACCACCCTGTTGCTGTA-3′ (antisense primer). A thermocycling program was set for 40 cycles of 3 s at 95°C, and 30 s at 60°C with an initial denaturation step at 95^o^C for 20 s. Gene expression values were normalized to endogenous control GAPDH, and calibrated to sample with the lowest expression. The 2^−ΔΔCt^ method was used to calculate fold changes in the gene expression normalized to GAPDH [16]. Relative quantification (RQ) = 2^-ΔΔCt^, (ΔΔCt = ΔCt sample - ΔCt calibrater, where ΔCt = Ct target gene - Ct GAPDH) [17].

### 2.10 Cytotoxicity Assay

Human malignant meningioma cell line IOMM-Lee cells were seeded at a density of 5 × 10^3^/well in a 96-well plate and cultured for 4 hours at 37°C. The IOMM-Lee cells were then incubated with 200 μl culture media containing anti-CD47 antibody (B6H12), IgG1 isotype control and PBS for 24 hours, respectively. The cell counting kit assay (KeyGEN BioTECH, CHINA) was used to measure cell proliferation [18]. Briefly, according to the manufacturer's instructions, 10 μl Cell-Counting Kit-8 (CCK-8) reagent was added into each well, and the optical density (OD) at 450 nm was recorded using a microplate reader (BioTek, USA) after incubation for 3 hours. The cell viability (%) was calculated using ((ODT- ODB) / (ODC-ODB)) × 100%. The experiment was carried out three times independently.

### 2.11 Migration Assay

Cell invasion assay was performed with self-coated Matrigel (BD Biosciences, San Jose, CA, USA) on the upper surface of a transwell chamber. The IOMM-Lee cells were planted in transwell chambers which were then put in a 12-well plate. The upper chambers were added with the cell suspension suspended with serum-free DMEM medium while the lower chambers were added the DMEM medium containing 10% FBS. The IOMM-Lee cells were incubated in a 5% CO_2_ incubator at 37°C for 24 hours. Anti-CD47 antibody (B6H12) at the concentration of 10 μg/ml was added to the upper chamber of the experimental group. IgG1 isotype control at the concentration of 10 μg/ml was added to the upper chamber of the control group. PBS at the same concentration was added to the upper chamber of the blank control group. After 24 hours, the medium was discarded. When cells had invaded through the extracellular matrix layer to the lower surface of the membrane, cells were fixed with 4% paraformaldehyde (PFA) for 30 minutes in PBS and stained with crystal violet for 15 minutes. The cells in each group were finally counted and photographs of four randomly selected fields of the fixed cells were captured under the microscope. The experiment was repeated three times independently.

### 2.12 Scratch Assay

Approximately 5 × 10^5^ IOMM-Lee cells were seeded per well in a six-well plate. After the cell density were about 100%, A 200 μl pipette tip was used to make a scratch vertically at the center of each well, perpendicular to the horizontal line. Afterwards, the cells were washed three times with PBS to washed away the streaked cells. Then, the anti-CD47 antibody (B6H12) at a concentration of 10 μg/ml prepared in 2ml DMEM medium were added in each well of the experimental group while IgG1 isotype control at the same concentration were added in the control group. Blank control group only contained 2 ml DMEM medium. The cells were incubated in a 0.5% CO_2_ incubator at 37°C. We observed and captured photographs of cells in each group every 6 hours until 48 hours after the treatment under the microscope [19].

### 2.13 *In Vivo* Test in Mice and Tumor Measurement

The cells in the logarithmic phase were digested with 0.25% trypsin and the supernatant was removed by centrifugation. 100 μl of the cell suspension was slowly injected into the flanks of 4 weeks old nude mice with 5 × 10^6^ cells per mouse using 1.0 ml syringe to form a subcutaneous mound [20]. All mice were subcutaneously injected with meningioma cells, and all tumor-forming mice were equally divided into six groups with six mice in each group: Negative control group, IgG1 isotype control group, Anti‐CD47 (B6H12) group, Anti‐CD47 (MIAP301) group, Negative control + Clodronate group and Anti‐CD47 (MIAP301) + Clodronate group. Anti-CD47 antibody (B6H12 or MIAP301) was injected intraperitoneally into the mice in the corresponding group, and IgG1 isotype control was injected into mice in the control group. The same amount of PBS was injected into the negative control group. Mouse macrophages were depleted by injection of clodronate-containing liposomes (FormuMax Scientific). An initiation dose of 200μl of clodronate liposomes was injected intraperitoneally into nude mice two days before the subcutaneously injection of 5×10^6^ of meningioma cells. To prevent the repopulation of macrophages, the mice were repeatedly injected with 100μl of clodronate liposomes every five days. Macrophage depletion was maintained throughout the experimental period. Starting 3 days after tumor cell injection, mice were treated with 100 µg of anti‐CD47 antibody (B6H12), anti‐CD47 antibody (MIAP301), IgG1 isotype control or PBS per day, respectively. Earrings were labeled to mark the mice [21]. The life status of nude mice in each group was checked regularly every day. The body mass of nude mice in subcutaneous tumor group was weighed every week. The curve of the tumor volume was drawed by the formula V = (π × width × length × height) / 6. After 5 weeks, mice were sacrificed and tumors were excised, measured and weighed.

### 2.14 Immunofluorescence Assay

Co-cultured cells were fixed with 4% PFA at 4°C for 15 minutes. After washing the cells with PBS, 0.3% Triton X-100 was added into the cells for 10 minutes. Co-cultured cells were then blocked with 10% goat serum (Vector Laboratories, Inc, Burlingame, CA, USA) for 1 hour at room temperature and incubated with primary antibodies overnight at 4°C. The primary antibody was CD68 antibody. The cells were then incubated with anti-rabbit IgG fluorescein-conjugated secondary antibody (1:200; Life Technologies) at room temperature for 1 hour and followed by nuclear counterstaining with 4-6-diamidino-2-phenylindole (DAPI, Abcam). The samples were detected by confocal microscopy. In all of the slices, five fields per sample and three to six tissues were quantified in each group. The expression was analyzed using Image-Pro Plus 6.0 software (Media Cybernetics Inc, Buckinghamshire, UK).

### 2.15 *In vitro* Phagocytosis Assay

Meningioma cells were labeled with 1μM CFDA-SE (CFSE) using the Cell Trace CFSE Cell Proliferation Kit (Invitrogen). Macrophages were incubated with PMA for 48 hours. Then macrophages were incubated with 1×10^6^ CFSE-labeled tumor cells in serum-free medium in the presence of 10μg/ml anti-CD47 antibody (B6H12; BioXcell) or 10μg/ml IgG1 isotype control (BioXcell) or PBS for 2hours, respectively. The macrophages were incubated with CD68 antibody to make macrophages red fluorescent protein (RFP) positive before observation. Immunofluorescence assay was performed to observed the phagocytosis. The phagocytic index was calculated as the number of phagocytosed CFSE^+^ cells per 100 macrophages.

### 2.16 Transfection

The IOMM-Lee cells were digested to form a single-cell suspension and were divided into three groups including short interfering (si)-CD47, scrambled si-RNA (si-NC) and control group. The cells were then planted in 6-well plates until the cells entered the logarithmic growth phase. When the cell density reached 70~80%, 20 nM si-CD47 or a scrambled si-RNA (si-NC) was transfected into the cells using Lipofectamine 2000 (Thermo Fisher Scientific, Inc.) according to the manufacturer's protocols. The sequences of siRNAs are: si-CD47, 5′-AGAUUUGACUUUACUAAGC-AG-3′ and si-NC, 5′-UUCUCCGAACGUGUCACGUTT-3′. After 24 hours of transfection, the mRNA expression of CD47 was determined by reverse qPCR.

### 2.17 Statistical Analysis

Statistical Analysis was performed using GraphPad Prism 5.0 (GraphPad software). All the data were analyzed by SPSS 19.0 software. The quantitative data was presented as mean ± SEM. The χ^2^ test was used to analyze the difference of immune checkpoint and infiltrated immune cells expression rate in different grades of meningioma. The one-way ANOVA test was used to analyze the difference of immune checkpoint expression intensity in different grades of meningioma and the differences between anti-CD47 group, NC group and IgG1 isotype control group. Overall survival analysis was performed using Kaplan-Meier analysis. All experiments were repeated three times.

## 3 RESULTS

### 3.1 The Expression of CD47 in Tumor Cells is Closely Related to the Malignant Grade of Meningioma

**Fig. (1):**
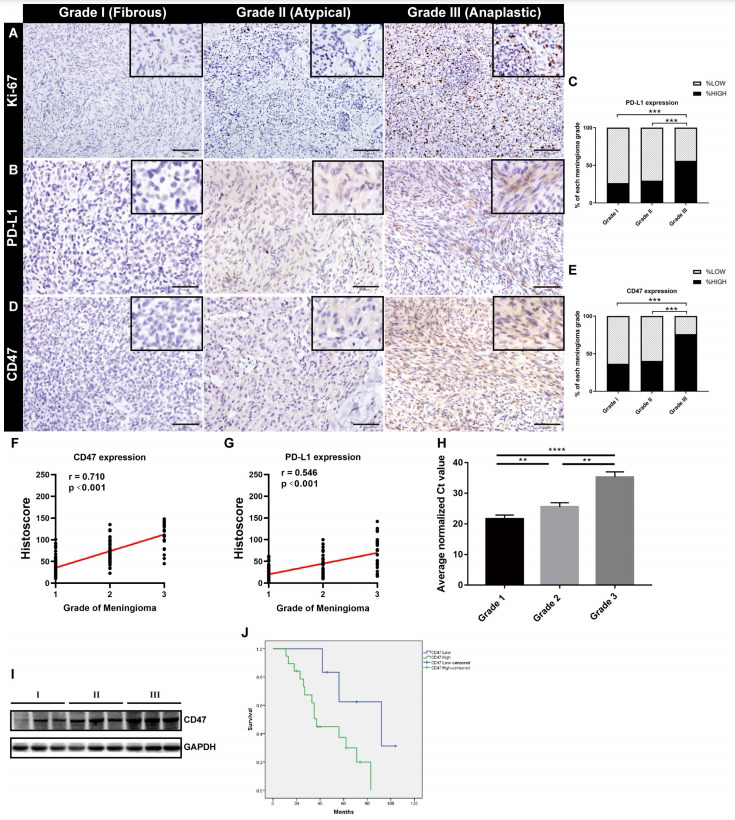
The expression analyses of CD47, PD-L1 and Ki-67 in grade I, II, III meningioma. (**A**, **B**, **D**) Representative images of immumohistochemical staining of Ki-67 (**A**), PD-L1 (**B**) and CD47 (**D**) in meningioma. (**C**, **E**) Analysis of the correlation between the expression rate of CD47, PD-L1 and the pathological grade of meningioma. (**F** and **G**) Analysis of the correlation between the expression intensity of CD47, PD-L1 and the pathological grade of meningioma. (**H** and **I**) qPCR analysis (**H**) and western blot analysis (**I**) of CD47 expression in primary meningioma cells in three grades meningioma (n=3/group). (**J**) Kaplan-Meier survival curves for malignant meningioma patients comparing those with CD47 expression higher than the mean and those CD47 expression lower than the mean. **p < 0.01, ***p < 0.001, ****p < 0.0001.

In this study, a total of 190 meningioma samples was collected in our cohort including 128 WHO grade I, 37 WHO grade II and 25 WHO grade III, with Ki-67 was highly expressed in 17.97% (23/128) of WHO grade I, 51.35% (19/37) of WHO grade II and 88.00% (22/25) of WHO grade III meningioma (Fig. **1A**, Tables **1** and **2**). To test the possibility of immunotherapy in meningioma treatment, we first detected the expression levels of immune checkpoints CD47 and PD-L1 in meningioma samples by using immunohistochemistry. The results showed that PD-L1 was mainly expressed in cell membrane, with 26.56% (34/128) of WHO grade I, 29.73% (11/37) of WHO grade II and 56.00% (14/25) of WHO grade III was highly expressed (Figs. **1B** and **C** and Table **2**). While CD47 was expressed in a cytoplasmic and membranous manner, with 36.72% (47/128) of WHO grade I, 40.54% (15/37) of WHO grade II and 76.00% (19/25) of WHO grade III was highly expressed (Figs. **1D** and **E** and Table **2**). Although the expression levels of PD-L1 and CD47 increased with the malignant degree of meningioma, the expression level of CD47 was higher than that of PD-L1 in all grades of meningioma (Figs. **1C**, **E**-**G** and Table **2**). Moreover, the expression intensity of CD47 in malignant meningioma (WHO grade III) was similar to that of breast cancer and lung cancer, which was considered to be highly expressed (Figs. **S1A** and **C**). Meanwhile, Dual immunofluorescence staining of malignant meningioma showed that CD47 was mainly expressed on the membrane of EMA-positive tumor cells (Fig. **S1D**). To further verify the expression level of CD47 in each grade of meningioma, qPCR and Western blot was performed and the results showed that the mRNA expression and protein expression levels of CD47 in WHO grade III meningioma were higher than those in WHO grade I and WHO grade II meningioma (Figs. **1H** and **I**). Finally, Kaplan-Meier survival analysis also showed that the overall survival in CD47-low group was better than that in CD47-high group of malignant meningioma (*P* = 0.035; Fig. **1J**).

Collectively, these results suggested that CD47 was highly expressed in meningioma and was closely related to the prognosis of meningioma, which maybe serve as a potential therapeutic target of meningioma.

### 3.2 Characterization of the Immune Cell Infiltration in Meningioma Tissues

**Fig. (2):**
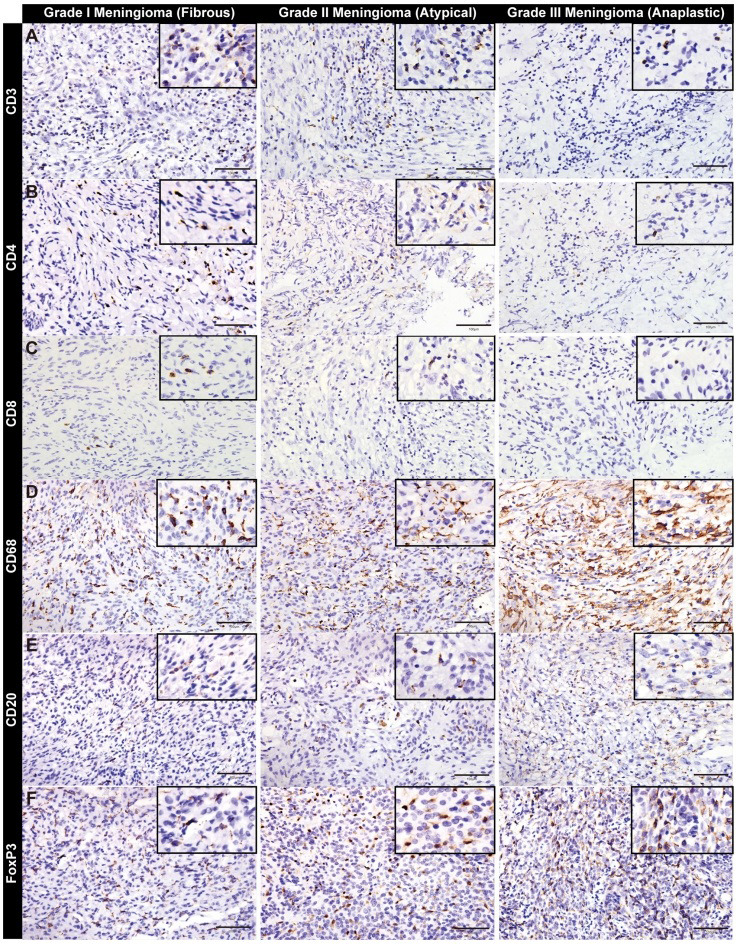
Immune cell infiltration analysis in grade I, II and II meningioma tissues. (**A-F**) Representative images of immumohistochemical staining of CD3 (**A**), CD4 (**B**), CD8 (**C**), CD68 (**D**), CD20 (**E**) and FoxP3 (**F**) in meningioma. The immune cell specific markers used include CD3, CD4, CD8 (T cells), CD68 (macrophages), CD20 (B cells), and FoxP3 (Regulatory T cells).

To detect the infiltration of immune cells in three grades of meningioma tissues, immunohistochemistry was performed and the results showed that the infiltrated immune cells in WHO grade III meningioma was mainly composed of CD68^+^ macrophages (*P* = 0.006) accompanied by a significant decrease of CD3^+^ (*P* = 0.008), CD4^+^ (*P* = 0.003) and CD8^+^ (*P* = 0.038) T lymphocytes compared with WHO grade I and WHO grade II meningioma (Fig. **2A**-**D** and Table **3**). Nevertheless, the infiltration of CD20^+^ B lymphocytes in WHO grade III meningioma (*P* = 0.866) was comparable to that of WHO grade I and WHO grade II meningioma (Fig. **2E** and Table **3**). Meanwhile, the expression of FoxP3 in WHO grade III meningioma (*P* < 0.001) was significantly higher than that of WHO grade I and WHO grade II meningioma (Fig. **2F** and Table **3**), indicating the regulatory T (Treg) cells were rich in malignant meningioma. We next carried out immunofluorescence staining in malignant meningioma tissues and showed that CD68^+^ macrophages were the major types of the immune cells compared with other immune cells (Fig. **S2A-C**), which was consistent with the results of immunohistochemistry analysis in mouse subcutaneous xenografts derived from the human malignant meningioma cell line IOMM-Lee (Figs. **S2D-L**).

Taken together, these results demonstrated the limitations on immunotherapy associated with PD-L1 which worked with T lymphocytes, and it also raised the possibility of CD47 as a potential therapeutic target for malignant meningioma, since macrophages are significantly enriched in malignant meningioma.

### 3.3 CD47 Blockade Inhibits the Proliferation, Migration and Invasion of Malignant Meningioma Cells *in vitro*

**Fig. (3):**
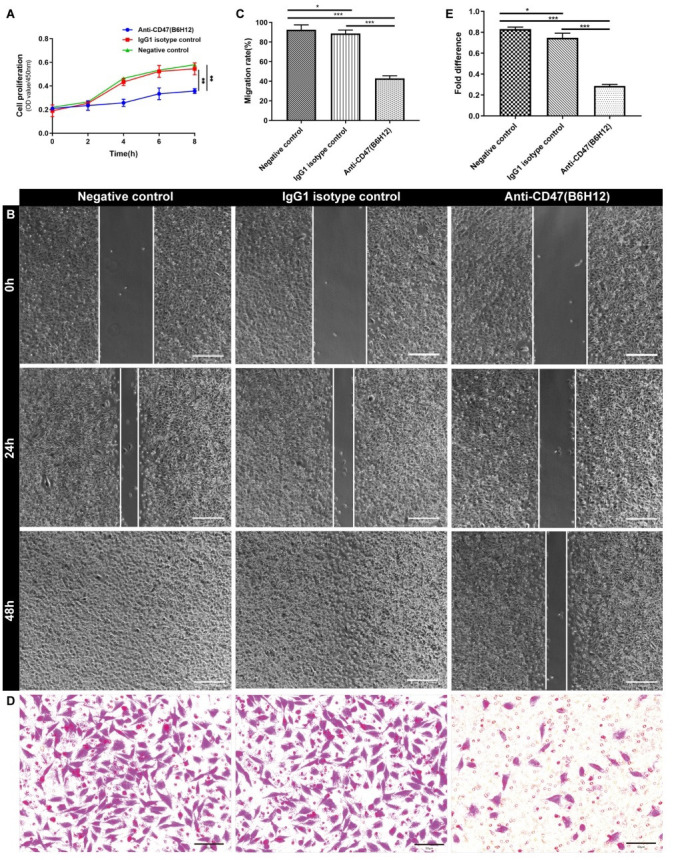
The effect of anti-CD47 antibody (B6H12) on the proliferation, migration and invasion ability of IOMM-Lee cells. (**A**) CCK-8 proliferation assay was used for detecting the proliferation ability of IOMM-Lee cells in the presence of 10 μg/mL anti-CD47 antibody (B6H12). (**B-E**) Anti-CD47 antibody (B6H12) significantly attenuated cell migration and invasion ability in IOMM-Lee cells as measured by wound healing assay (**B**) and transwell invasion assay (**D**). Graphical illustration of statistical results of anti-CD47 on cell migration (**C**) and invasion (**E**). *p < 0.05, ***p < 0.001.

Previous studies have been suggested that CD47 affected the biological behaviour like proliferation, migration and invasion in various tumor types. We have verified previously that CD47 was highly expressed in malignant meningioma. Therefore, we tested whether CD47 influenced the proliferation, migration and invasion of malignant meningioma cells *in vitro*. The human malignant meningioma cell line IOMM-Lee cells were firstly treated with or without anti-CD47 antibody (B6H12) or IgG1 isotype control. The results of CCK-8 assay demonstrated that the proliferation of IOMM-Lee cells treated with B6H12 was significantly decreased compared with IOMM-Lee cells treated with IgG1 isotype control or cells in the negative control (NC) group (Fig. **3A**). Next, the cell scratch assay was performed to detect the effect of CD47 on cell migration. Indeed, the wound healing ability of IOMM-Lee cells in anti-CD47 group could be significantly attenuated (Fig. **3B** and **C**). Finally, the transwell invasion assay with matrigel was performed to assess the role of CD47 on the invasion ability of IOMM-Lee cells. The results suggested that CD47 promoted the invasive ability of IOMM-Lee cells (Fig. **3D** and **E**).

**Fig. (4):**
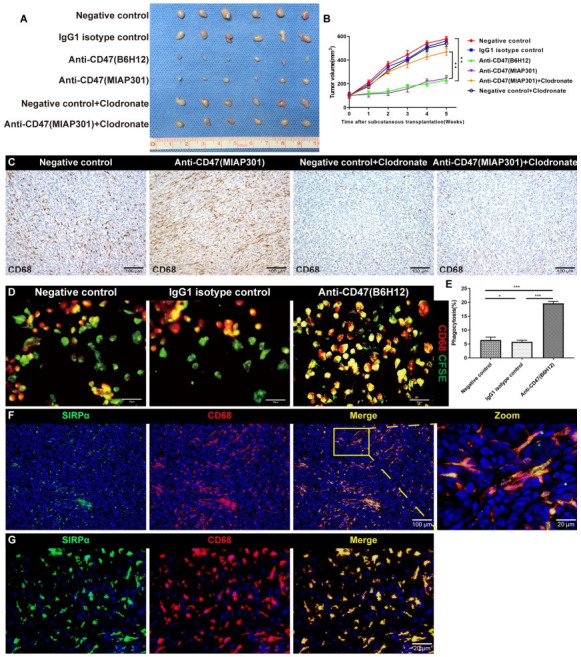
Nude mouse models bearing subcutaneous meningioma and *in vitro* phagocytosis assay. (**A**) Representative images of the gross tumors of each group are shown. (**B**) Tumor growth curves were measured during the growth of the tumors in the indicated groups. (**C**) Representative images of immumohistochemical staining of CD68 showing the efficiency of macrophage depletion *in vivo* by using clodronate-containing liposomes. (**D**) The macrophages were co-cultured with IOMM-Lee cells in the presence of IgG1 isotype control or anti-CD47 antibody (B6H12). Representative overlay images (one section of a field of view) for each condition is shown. IOMM-Lee cells were labelled with CFSE (green) and macrophages were labelled with CD68 (red). Yellow indicates macrophages have phagocytosed IOMM-Lee cells. (**E**) Quantification analysis shows the number of CD68^+^CFSE^+^ cells in anti-CD47 antibody group is significantly higher than that of control and IgG1 isotype control group. (**F** and **G**) Double immunofluorescence staining of mouse subcutaneous meningioma (**F**) and human meningioma tissue samples (**G**); representative images showing the co-expression of SIRPα and CD68. Boxed area is enlarged and presented on the right. *p < 0.05, ***p < 0.001.

To validate the potential impact of CD47 blockade on IOMM-Lee cell proliferation *in vivo*, the IOMM-Lee cells were injected subcutaneously in nude mice and the mice with established tumors were randomized into six groups with treatment with or without anti-CD47 antibody (B6H12), anti-CD47 antibody (MIAP301), IgG1 isotype control or clodronate-containing liposomes, respectively. Tumors in mice treated with B6H12 and MIAP301 grew slower and exhibited significantly smaller tumor volume 5 weeks after injection in comparison with IgG1 isotype control group cells or NC group cells (Figs. **4A** and **B**). However, the inhibition effect of MIAP301 on tumor growth was partly blocked by the depletion of macrophages (Fig. **4A**-**C**).

Therefore, combined with the results mentioned above, we proposed that CD47 could enhance the cell growth, migration and invasion of IOMM-Lee cells.

### 3.4 Anti-CD47 Induces Macrophage-mediated Phagocytosis in Malignant Meningioma

It has been reported that tumor cells co-cultured with macrophages under the intervention of anti-CD47 antibody could promote the tumor recognition and phagocytosis of macrophages [22]. Thus, To verify that CD47 blockade would promote the phagocytosis of malignant meningioma cells by macrophages, malignant meningioma cells and macrophages were co-cultured with or without the presence of anti-CD47 antibody (B6H12) or IgG1 isotype control. The results of immunofluorescence staining suggested that the phagocytosis rate of macrophages was significantly higher in the anti-CD47 group compared with the IgG1 isotype control or NC groups (Figs. **4D**-**E**). Moreover, we examined the expression of signal regulatory protein-alpha (SIRPα), one of the most important receptor of CD47, in both malignant meningioma tissues and mouse subcutaneous xenografts derived from the human malignant meningioma cell line IOMM-Lee. Results revealed that SIRPα was co-expressed with CD68 both in malignant meningioma tissues and mouse subcutaneous xenografts, indicating that the phagocytosis of malignant meningioma cells by macrophages maybe mediate by CD47-SIRPα axis (Figs. **4F** and **G**).

These results demonstrated that CD47 blockade could promote the phagocytosis of malignant meningioma cells by macrophages. Therefore, CD47 may mediate the immune escape of malignant meningioma cells *via* CD47-SIRPα interaction.

### 3.5 CD47 Participates in Multiple Signaling Pathways to Affect Meningioma Progression

**Fig. (5):**
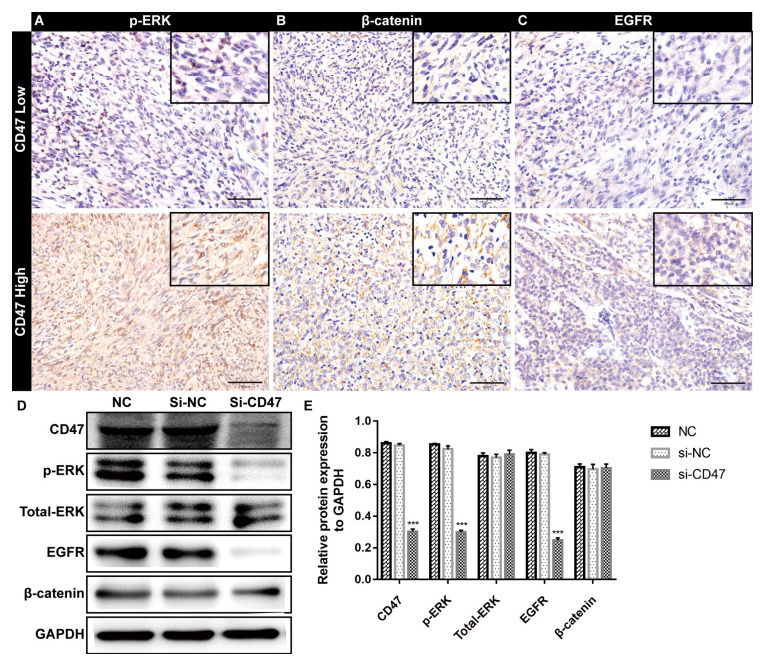
CD47 related signaling pathway analysis in grade III meningioma. (**A-C**) Representative images of immumohistochemical staining of p-ERK (**A**), β-catenin (**B**) and EGFR (**C**) in CD47-high and CD47-low meningioma of grade III. (**D-E**) Western blot analysis showed that the expression of CD47 in IOMM-Lee cells transfected with si-CD47 was significantly reduced, also along with a significant decrease in p-ERK and EGFR expression compared with si-NC and control group. ***p < 0.001.

Various signaling pathways associated with CD47 have been extensively studied [1]. However, CD47 associated signaling pathways in malignant meningioma are still limited. In light of this, the expression of EGFR, p-ERK and β-catenin in meningioma tissues from WHO grade I, II and III was examined by immunohistochemistry. The results showed that p-ERK, EGFR and β-catenin were expressed in all grades of meningioma with the highest expression levels in WHO grade III meningioma (Figs. **5A**-**C**). To further verify the potential role of CD47 in malignant meningioma, siRNA that targeted to CD47 were designed to silence its expression in malignant meningioma cells. The results indicated that the expression of CD47 in malignant meningioma cells was significantly inhibited in si-CD47 group compared with si-NC and NC groups (Fig. **5D** and **E**). Along with that, the expression of p-ERK and EGFR was also remarkablely down-regulated in si-CD47 group cells, indicating the MAPK and EGFR signaling pathways were regulated by CD47 in malignant meningioma cells.

## 4 DISCUSSION

At present, many studies have reported the progress of immunotherapy in treating multiple tumor types [23]. The increased expression of the classical immune checkpoint PD-L1 has been reported in meningioma [14]. Furthermore, the expression of PD-L1 was also associated with the increased tumor aggressiveness [24]. Multiple PD-L1 inhibitors are showing good consequences in treating other tumors [25]. However, the results of immunohistochemistry suggested that compared with PD-L1, the expression of CD47 was higher in malignant meningioma which indicated CD47 blockade maybe more effective in treating malignant meningioma. The expression of CD47 has been reported in many tumors such as breast cancer, thyroid cancer, and colorectal cancer [7-9]. CD47 provides a “don’t eat me” signal by binding to SIRPα [6]. Blocking CD47 with antibody could turn off the “don't eat me” signal and favor phagocytosis of macrophages [11, 26]. Surprisingly, our results showed that the expression of CD47 was higher in WHO grade III meningioma than that of grade I and II. Further, the detection of the infiltrated immune cells in meningioma tissues showed that there was a decreased expression of CD3^+^, CD4^+^ and CD8^+^ T lymphocytes in malignant meningioma, which implied PD-L1 blockade may not have outstanding effect for malignant meningioma therapy. Previous studies have demonstrated the therapeutic effect of CD47 blockade requires functional dendritic cells (DCs) [27]. Meanwhile, we found that CD68^+^ macrophages showed an increased expression in malignant meningioma, which indicated blocking CD47 maybe a potential therapeutic target for malignant meningioma.

Previous studies have shown that anti-CD47 inhibited the proliferation, invasion and migration of tumor cells [8, 10, 13]. However, no one has reported the effect of anti-CD47 on malignant meningioma. So we conducted the experiments above and found that anti-CD47 antibody (B6H12) could indeed inhibit the proliferation, migration and invasion ability of malignant meningioma. Furthermore, we tested the effect of anti-CD47 by using two different clones of anti-CD47 antibody (B6H12 and MIAP301) *in vivo* and the results showed that both B6H12 and MIAP301 could inhibit the progression of meningioma, which was consistent with the findings of previous study on other tumors. Additionally, the anti-tumor effect of MIAP301 was found to be partly blocked under the condition of macrophage depletion, as anti-CD47 itself could not only promote the phagocytosis of macrophages to meningioma, but also inhibit the proliferation and migration of meningioma.

CD47 on the tumor surface produces corresponding signals by binding to SIRPα on macrophages, which makes macrophages unable to recognize and phagocytize tumor cells [28]. Tumor cells and macrophages were co-cultured and treated with anti-CD47 antibody (B6H12) or IgG1 isotype control in this study. Our results showed that the activation and phagocytosis rate of macrophages in anti-CD47 group were significantly higher than that of control group, which confirmed the suggestion above.

According to previous studies, there were multiple signaling pathways related to CD47 [29, 30], including EGFR, MAPK and WNT signaling pathways [31-33]. Here, we showed that p-ERK, EGFR was highly expressed in malignant meningioma compared to β-catenin. Furthermore, the expression of EGFR was higher in CD47 high expression group, which suggesting CD47 may affect the progression of meningioma by affecting EGFR pathway. It is worthy noting that meningioma cells are capable of autocrine expression of the EGFR ligands, such as TGF-α and EGF [1]. Previous studies suggested that anti-CD47 antibody down-regulated EGFR expression at the mRNA and protein levels [31]. These results demonstrated that therapeutic CD47 antibodies may be effective against tumors with high EGFR expression when used alone or in combination with EGFR inhibitors, which maybe a novel treatment direction for malignant meningioma. However, in clinical trials, anemia and thrombocytopenia have become the dose-limiting toxicity of CD47 because it is also involved in the clearance of red blood cells (RBCs) in the human body. More importantly, the large number of RBCs in the body will be the best cover for tumor cells, as drugs targeted to CD47 will be depleted by RBCs before they reach the tumor cells. Therefore, the application of drugs targeting CD47 in clinical practice still needs further study.

## CONCLUSION

To sum up, we should realize that CD47 blockade plays a practical and effective role in eliminating tumor immune escape and inhibiting the proliferation and migration of malignant meningioma. Meanwhile, we should also consider that the core of the development of anti-CD47 drugs is how to kill tumor cells to the maximum extent while protecting RBCs from accidentally injure [34]. It is believed that with the development of targeting technology and drug targeting carriers, anti-CD47 drugs will be targeted and quantitatively acted on local tumor tissues, especially tumors such as malignant meningioma, so as to achieve the effective cure of tumors.

## Figures and Tables

**Table 1:** Clinicopathological characteristics of meningioma parents in our cohort.

**Clinical Parameters**	**Entire Population (n = 190)**	
	**n**	**%**
**Meningioma Subtype**		
***WHO Grade I***	***128***	-
Meningothelial	25	19.5
Fibrous	32	19.6
Transitional	61	47.7
Angiomatous	6	4.5
Psammomatous	3	2.3
Metaplastic	1	1.0
***WHO Grade II***	***37***	-
Atypical	29	78.4
Chordoid	8	21.6
***WHO Grade III***	***25***	-
Anaplastic	16	64.0
Rhabdoid	5	20.0
Papillary	4	16.0
**Age**		
Median	51	-
Range	13-82	-
15-44	55	28.9
45-59	78	41.1
60-100	57	30.0
**Gender**		
Male	68	35.8
Female	122	64.2

**Table 2:** The expression of immune checkpoints CD47 and PD-L1 in 190 cases of meningioma.

**Pathological**	**Cases**	**CD47**			***P-value***	**PD-L1**			***P-value***	**Ki-67**			***P-value***
**Types**		**Low**	**High**	**Percent**		**Low**	**High**	**Percent**		**Low**	**High**	**Percent**	
***WHO Grade I***	***128***	***81***	***47***	***36.72%***	0.001	***94***	***34***	***26.56%***	0.004	***105***	***23***	***17.97%***	*<0.001*
Meningothelial	25	16	9	-	-	14	11	-	-	21	4	-	-
Fibrous	32	20	12	-	-	23	9	-	-	26	6	-	-
Transitional	61	37	24	-	-	51	10	-	-	50	11	-	-
Angiomatous	6	5	1	-	-	4	2	-	-	5	1	-	-
Psammomatous	3	2	1	-	-	2	1	-	-	2	1	-	-
Metaplastic	1	1	0	-	-	0	1	-	-	1	0	-	-
***WHO Grade II***	***37***	***22***	***15***	***40.54%***	0.015	***26***	***11***	***29.73%***	0.039	***18***	***19***	***51.35%***	*0.003*
Atypical	29	17	12	-	*-*	22	7	-	-	16	13	-	*-*
Chordoid	8	5	3	-	*-*	4	4	-	-	2	6	-	*-*
***WHO Grade III***	***25***	***6***	***19***	***76.00%***	*-*	***11***	***14***	***56.00%***	-	***3***	***22***	***88.00%***	*-*
Anaplastic	16	1	15	*-*	*-*	***5***	***11***	*-*	*-*	1	15	*-*	*-*
Rhabdoid	5	2	3	*-*	*-*	***4***	***1***	*-*	*-*	2	3	*-*	*-*
Papillary	4	3	1	*-*	*-*	2	2	*-*	*-*	0	4	*-*	*-*

**Note**: The expression of CD47, PD-L1 and Ki-67 was separated into low and high expressors-relative to the median score for each marker for the entire cohort; specimens were classified according to pathological types. The percentage of cases with low or high infiltration are presented for each marker (< or > the median score across all of the samples in the cohort for each marker, respectively).

**Table 3:** The expression of CD3, CD4, CD8, CD68, CD20 and FoxP3 in 190 cases of meningioma.

**Pathological**	**Cases**	**CD3**			***P-value***	**CD4**			***P-value***	***CD8***			***P-value***	**CD68**			***P-value***	**CD20**			***P-value***	**FoxP3**			***P-value***
**Types**		**Low**	**High**	**Percent**		**Low**	**High**	**Percent**		**Low**	**High**	**Percent**		**Low**	**High**	**Percent**		**Low**	**High**	**Percent**		**Low**	**High**	**Percent**	
***WHO*** ***Grade I***	***128***	***50***	***78***	***60.93%***	*0.008*	***55***	***73***	***57.03%***	*0.003*	***53***	***75***	***58.59%***	*0.038*	***52***	***76***	***59.38%***	*0.006*	***90***	***38***	***29.69%***	*0.866*	***88***	***40***	***31.25%***	*<0.001*
Meningothelial	25	10	15	-	-	8	17	-	-	11	14	-	-	16	9	-	-	11	14	-	-	18	7	-	-
Fibrous	32	8	24	-	-	10	22	-	-	13	19	-	-	22	10	-	-	26	6	-	-	16	16	-	-
Transitional	61	27	34	-	-	33	28	-	-	26	35	-	-	11	50	-	-	47	14	-	-	46	15	-	-
Angiomatous	6	2	4	-	-	1	5	-	-	2	4	-	-	2	4	-	-	3	3	-	-	5	1	-	-
Psammomatous	3	2	1	-	-	3	0	-	-	1	2	-	-	1	2	-	-	2	1	-	-	2	1	-	-
Metaplastic	1	1	0	-	-	0	1	-	-	0	1	-	-	0	1	-	-	1	0	-	-	1	0	-	-
***WHO*** ***Grade II***	***37***	***13***	***24***	***64.86%***	*0.025*	***18***	***19***	***51.35%***	*0.031*	***17***	***20***	***54.05%***	*0.07*	***14***	***23***	***62.16%***	*0.025*	***25***	***12***	***32.43%***	*0.710*	***18***	***19***	***51.35%***	*0.008*
Atypical	29	11	18	-	-	12	17	-	-	14	15	-	-	11	18	-	-	18	11	-	-	16	13	-	-
Chordoid	8	2	6	-	-	6	2	-	-	3	5	-	-	3	5	-	-	7	1	-	-	2	6	-	-
***WHO*** ***Grade III***	***25***	***16***	***9***	***36.00%***	***-***	***19***	***6***	***24.00%***	***-***	***17***	***8***	***32.00%***	***-***	***3***	***22***	***88.00%***	***-***	***18***	***7***	***28.00%***	***-***	***4***	***21***	***84.00%***	***-***
Anaplastic	16	10	6	-	-	12	4	-	-	11	5	-	-	1	15	-	-	13	3	-	-	1	15	-	-
Rhabdoid	5	2	3	-	-	4	1	-	-	3	2	-	-	1	4	-	-	3	2	-	-	2	3	-	-
papillary	4	4	0	-	-	3	1	-	-	3	1	-	-	1	3	-	-	2	2	-	-	1	3	-	-

## Data Availability

The datasets generated during and/or analyzed during the current study are available from the corresponding author on reasonable request.

## LIST OF ABBREVIATIONS

AKT: Protein Kinase B
CCK-8: Cell Counting kit-8
CD47: Cluster of Differentiation 47
CFSE: Carboxyfluorescein Diacetate, Succinimidyl Ester
DAB: 3’,3-Diaminobenzidine
DAPI: 4’,6-Diamidino-2-phenylindole
DCs: Dendritic Cells
DMEM: Dulbecco's Modified Eagle's Medium
DMSO: Dimethyl Sulfoxide
EDTA: Ethylenediamine Tetraacetic Acid
EGFR: Epidermal Growth Factor Receptor
FBS: Fetal Bovine Serum
FFPE: Formalin-fixed and Paraffin-Embedded
FoxP3: Forkhead Box Protein 3
GAPDH: Glyceraldehyde-3-phosphate Dehydrogenase
HE: Haematoxylin and Eosin
HPFs: High-power Fields
IHC: Immunohistochemistry
IOMM-Lee: IntraOsseous Malignant Meningioma-Lee
MAPK: Mitogen-activated Protein Kinase
OD: Optical Density
one-way ANOVA: One-way Analysis of Variance
p-ERK: Phospho-extracellular Regulated Protein Kinases
PBS: Phosphate Buffer Saline
PD-L1: Programmed Cell Death-ligand 1
PFA: Paraformaldehyde
PI3K: Phosphoinositide-3 Kinase
PMA: Phorbol 12-myristate-13-acetate
qPCR: Quantitative Polymerase Chain Reaction
RBCs: Red Blood Cells
RFP: Red Fluorescent Protein
SEM: Standard Error of Mean
SIRPα: Signal Regulatory Protein α
TAMs: Tumor-associated Macrophages
Total-ERK: Total-extracellular Regulated Protein Kinases
WHO: World Health Organization
