# Supplementary material for: Tumor-selective Blockade of CD47 Signaling with CD47 Antibody for Enhanced Anti-tumor Activity in Malignant Meningioma
Source: Curr Neuropharmacol. 2023 Aug 15;21(10):2159–73. doi: 10.2174/1570159X21666230511123157 (PMC10556363; doi:10.2174/1570159X21666230511123157)
Supplement: Supplementary file 1 [file CN-21-2159_SD1.pdf]

Supplementary Material

Tumor-selective Blockade of CD47 Signaling with CD47 Antibody for Enhanced Anti-tumor Activity in Malignant Meningioma

Xiaotong Liu[1,#], Huarong Zhang[2,#], Chaohu Wang[2,#], Zhiyong Li[2], Qianchao Zhu[2], Yiwen Feng[2], Jun Fan[2], Songtao Qi[2,*], Zhiyong Wu[3,*] and Yi Liu[2,*]

[1]Southern Medical University, Guangzhou, Guangdong, China; [2]Department of Neurosurgery, Nanfang Hospital, Southern Medical University, Guangzhou, Guangdong, China; [3]College of Traditional Chinese Medicine, Southern Medical University, Guangzhou, Guangdong, China

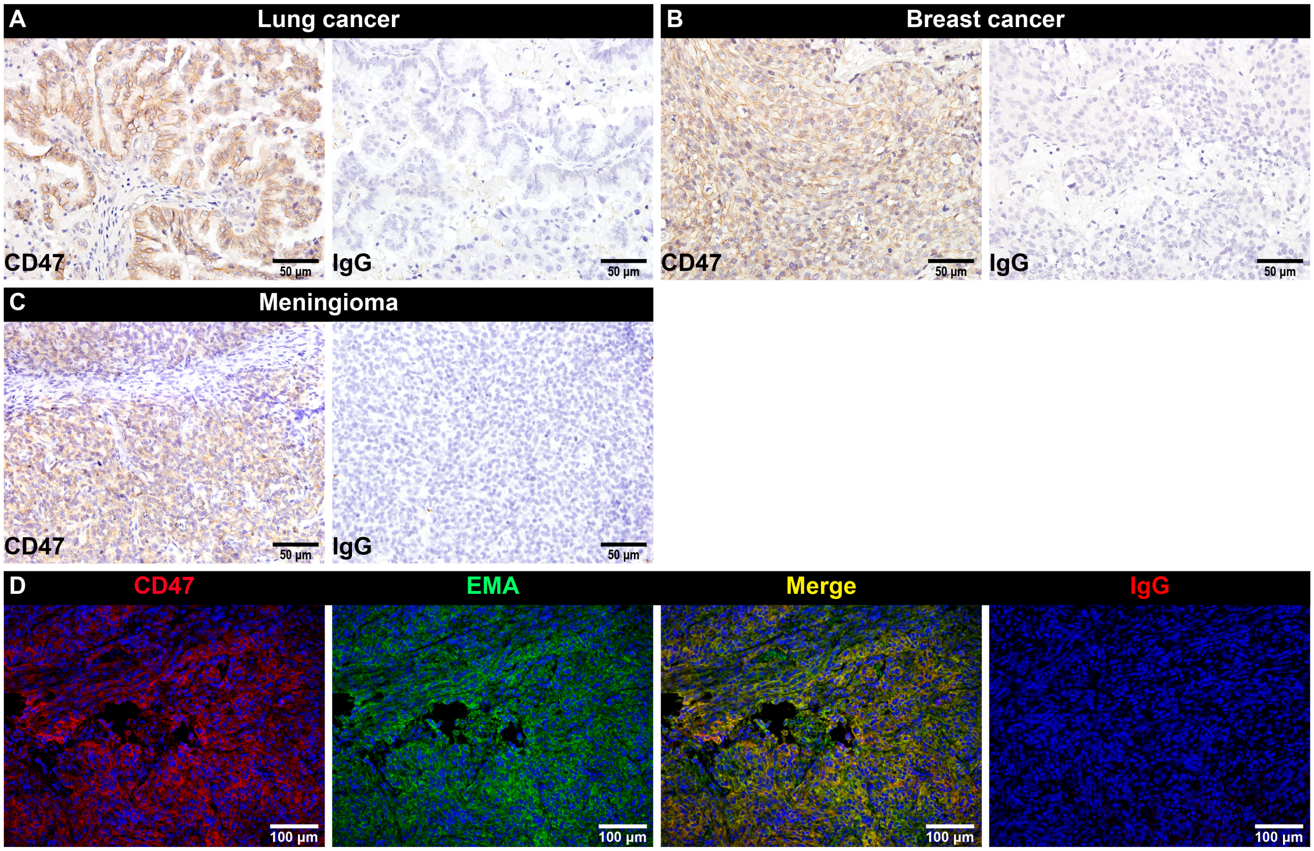

Figure S1 Expression of CD47 in tissue sections of human malignant meningioma with positive and negative controls. **(A–C)** Representative images of immumohistochemical staining of CD47 (left) and corresponding negative control (IgG, right) in human lung cancer tissues (A), human breast cancer tissues (B) and human malignant meningioma tissues (C). **(D)** Double immunofluorescence staining of human malignant meningioma tissue samples; representative images showing the expression of CD47 and EMA (marker of meningioma cells). The corresponding negative control (IgG) is presented on the right.

1875-6190/23 $65.00+.00 © 2023 Bentham Science Publishers

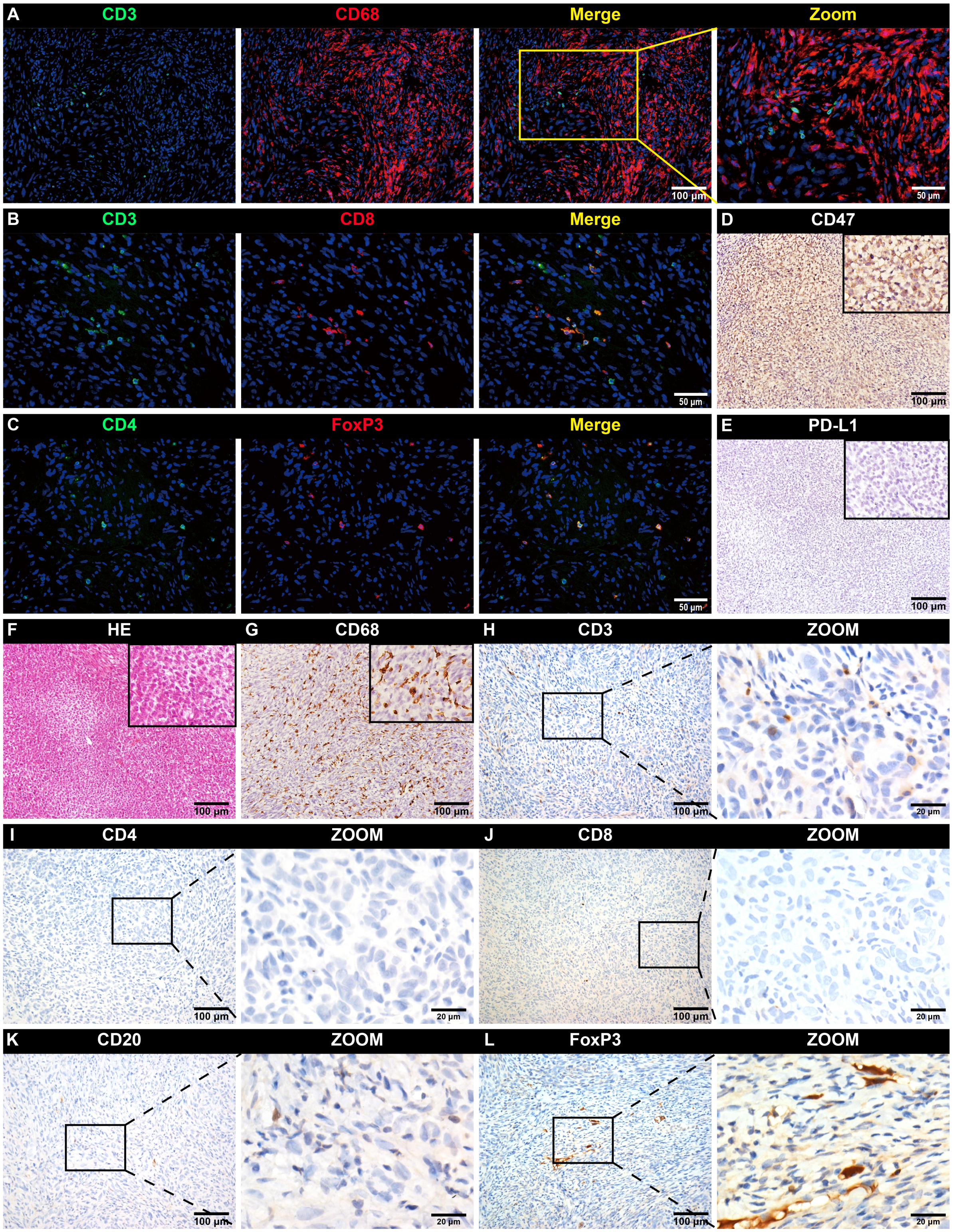

Figure S2 The immune cell infiltration in human malignant meningioma and mouse subcutaneous meningioma samples. **(A)** Immunofluorescence staining of human malignant meningioma tissue samples, representative images of CD3 and CD68 staining. Boxed area is enlarged and presented on the right. **(B)** Immunofluorescence staining of human malignant meningioma tissue samples, representative images of CD3 and CD8 staining. **(C)** Immunofluorescence staining of human malignant meningioma tissue samples, representative images of CD4 and FoxP3 staining. **(D–E)** Representative images of immumohistochemical staining of CD47 (D) and PD-L1 (E) in mouse subcutaneous meningioma tissue samples. **(F)** Representative images of HE staining of mouse subcutaneous meningioma tissue samples. **(G–L)** Representative images of immumohistochemical staining of CD68 (G), CD3 (H), CD4 (I), CD8 (J), CD20 (K) and FoxP3 (L) in mouse subcutaneous meningioma tissue samples. Boxed area is enlarged and presented on the right.
